# Study on Microstructure and Properties of Q460 Steel at Different Ni Contents

**DOI:** 10.3390/ma19040704

**Published:** 2026-02-12

**Authors:** Xuehai Qian, Weiping Lu, Zhen Li, Zecheng Zhuang, Lei Zeng, Jianping Tan

**Affiliations:** 1 School of Mechanical and Electrical Engineering, Central South University, Changsha 410083, China; 2 Technology Centre, Guangxi Liuzhou Iron and Steel Group Ltd., Liuzhou 545002, China

**Keywords:** Ni content, Q460 steel, impact energy, bainite

## Abstract

Q460 steel with varying Ni contents was produced via the hot continuous rolling process. The microstructure and properties were examined using scanning electron microscopy (SEM) and universal testing machines. The results show that with increasing Ni content, the number of inclusions rises, and the microstructure gradually evolves from ferrite and pearlite to a mixture of ferrite, pearlite, and bainite. Due to the formation of bainite and refined grains, both the yield strength and tensile strength are significantly improved. However, with the increase in inclusions, the elongation and impact energy of the material do not exhibit substantial enhancement. Considering both cost and performance, the Ni content in Q460 steel is preferably controlled at 0.3%.

## 1. Introduction

Q460 is a low-alloy high-strength structural steel, known for its excellent strength, good ductility and toughness, as well as favorable aspects of weldability [1,2,3,4,5]. It is currently widely used in high-rise buildings, bridge engineering, and various machines [4,5,6,7].

Given increasingly severe operating conditions, enhancing the mechanical properties of Q460 steel has become an urgent issue. A series of studies have been conducted by researchers. Meng et al. [8] investigated the influence of different process parameters on the toughness of Q460 steel, concluding that a two-stage rolling process should be adopted to ensure good toughness in Q460 plates. Chen et al. [9] examined the effects of different tempering processes on the microstructure and properties of Q460 plates. With increasing tempering temperature, coarsening of bainite plates was observed, along with a decrease in dislocation density. Wang et al. [10] studied the effects of controlled rolling and controlled cooling parameters on the microstructure and properties of Q460q steel. The results indicated that both yield strength and tensile strength increased when the final cooling temperature or the opening temperature of the non-recrystallization zone was lowered. Yuan et al. [11] focused on the impacts of annealing processes on the impact performance of Q460 steel. After annealing treatment, the impact energy of Q460 steel at −40 °C increased from 135 J to 254 J. Guo et al. [12] experimentally investigated the mechanical properties and creep behavior of Q460 using a Gleeble-3800 thermal simulation machine. Based on experimental data, a high-temperature creep constitutive equation was derived, which provided a basis for finite element simulations of hot-rolled Q460.

As evidenced by the works in the literature cited above, domestic and international research has primarily focused on rolling processes aiming to enhance the mechanical properties of Q460 steel, with limited investigation into the effects of chemical composition. While nickel, as a grain-refining element, can significantly improve material performance, its substantial addition inevitably increases production costs [13,14,15,16,17,18].

In this study, Q460 steel plates with varying nickel contents were prepared using the TMCP process. Through comparative analysis, the influence of nickel content on the microstructure and mechanical properties of Q460 steel is systematically examined, with the aim of determining the optimal nickel addition level for Q460 steel.

## 2. Materials and Methods

### 2.1. Chemical Composition

Three types of Q460 steel with different Ni contents were designed for this experiment. The steel was smelted in a 50 kg vacuum induction furnace and cast into ingots with dimensions of φ150 × 300 mm. The chemical compositions of the billets are presented in Table 1. To ensure a single-variable configuration, the three experimental steels were prepared with essentially identical elemental compositions except for the Ni content.

### 2.2. Rolling Processes

**Figure 1:**
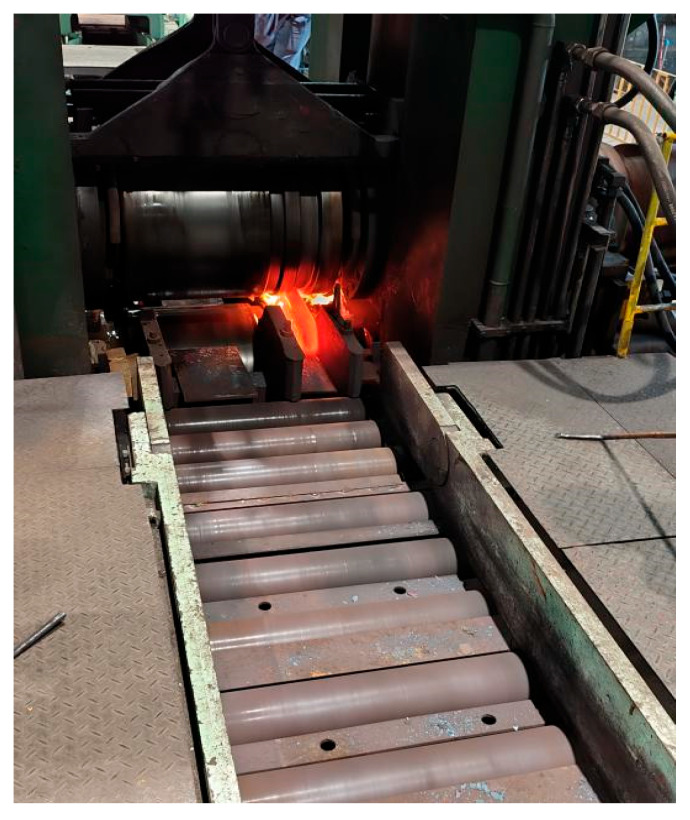
Photograph showing the rolling process of Q460 steel.

**Figure 2:**
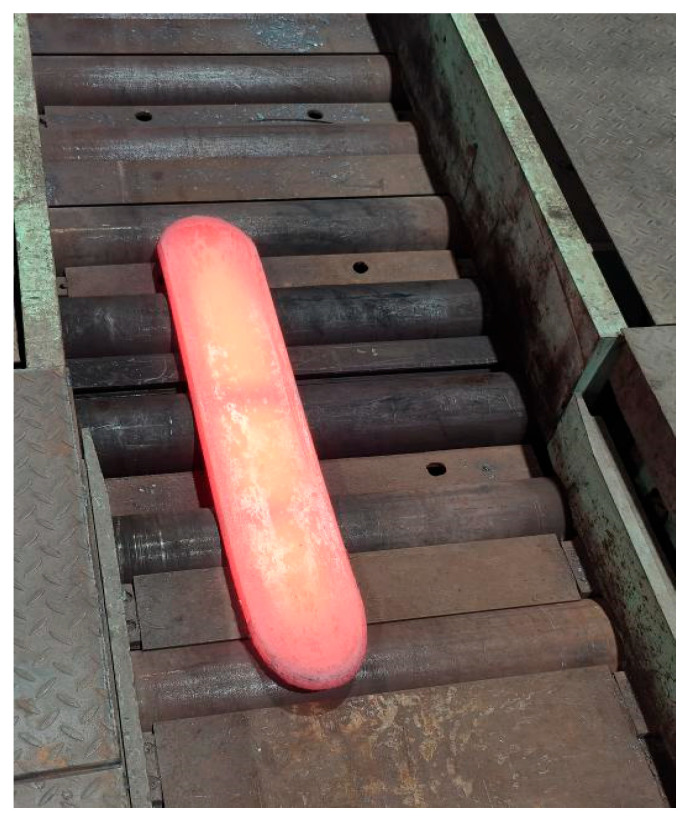
Photograph showing the shape of Q460 steel after rolling.

**Figure 3:**
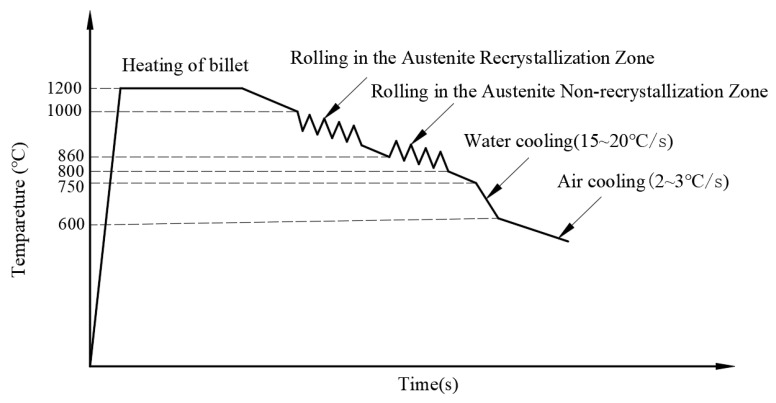
The temperature variation curve during the steel plate preparation process.

Hot rolling tests were conducted on a φ800 mm two-high reversible rolling mill, with the billet heated to 1200 °C and held for 2 h. A two-stage rolling process was employed, wherein the initial rolling temperature in the austenite recrystallization zone was 1000 °C, and this was followed by rolling in the austenite non-recrystallization zone, starting at 860 °C and finishing at 800 °C. After rolling, water cooling was immediately initiated at 750 °C until a return roll temperature of 600 °C was reached. The slab, with an initial thickness of 45 mm, was rolled in a total of seven passes to a final plate thickness of 20 mm, with the specific reduction per pass detailed in Table 2. The rolling schedule is illustrated in Figure 1, and the post-rolling configuration of the steel plate is shown in Figure 2. The temperature variation curve during the steel plate preparation process is shown in Figure 3.

### 2.3. Microstructure and Mechanical Properties

#### 2.3.1. Tensile Properties

**Figure 4:**
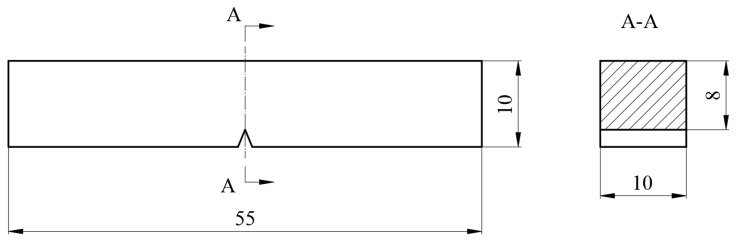
Schematic diagram showing the dimensions of the tensile specimens (wtmm).

In accordance with the relevant requirements of Chinese National Standard GB/T 1591-2018 [19], tensile property tests were conducted on the Q460 steel. The specific procedure is as follows: First, the steel plate was machined into standard tensile specimens. Then, these specimens were subjected to tensile testing using a universal testing machine (WEW-100, Jinan Shijin Co., Ltd., Wuxi, China). The dimensions of the tensile specimens are schematically illustrated in Figure 4.

#### 2.3.2. Impact Test

**Figure 5:**
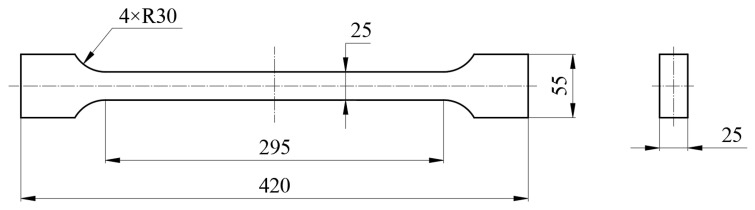
Schematic diagram of the impact specimen dimensions (wtmm).

In accordance with the relevant requirements of Chinese National Standard GB/T 1591-2018, a standard Charpy impact test was performed on the Q460 steel. The specific procedure is as follows: First, longitudinal impact specimens were extracted from the core region of the steel plate. The specimens’ dimensions were 10 mm × 10 mm × 55 mm with a 2 mm deep V-notch. Impact testing was then conducted using pendulum impact tester (JB-300B, Jinan Shijin Co., Ltd., Jinan, Shandong, China) at temperatures of −20 °C and −40 °C. A schematic diagram of the impact specimen dimensions is shown in Figure 5.

#### 2.3.3. Time-Dependent Impact Test

First, the steel plate was processed into standard tensile specimens. Then, these specimens were subjected to a 5% strain tensile test. Next, the specimens were heated to 250 °C ± 10 °C and held at that temperature for one hour before being air cooled to room temperature. Finally, an impact test specimen was cut from the center of each tensile specimen and impact testing was performed using a pendulum impact tester (JB-300B, Jinan Shijin Co., Ltd., Jinan, Shandong, China). The impact temperatures were −20 °C and −40 °C.

#### 2.3.4. Metallographic Analysis

First, the specimens were ground sequentially using 150-grit, 400-grit, 600-grit, 800-grit, 1200-grit, and 1500-grit sandpaper, followed by polishing. Then the specimens were etched with a 4% nitric acid–alcohol solution. Finally, the microstructures were examined under a metallurgical microscope (SZKESWAY, SZKESWAY Optical Instruments Co., Ltd., Shenzhen, Guangdong, China). After examination, the grain size was statistically analyzed according to the relevant requirements (area method) of Chinese National Standard GB/T 6394-2017 [20].

#### 2.3.5. Inclusion Analysis

Consistent with metallographic analysis, the specimens were first ground and polished and then examined using an electron probe (EPMA—8050G, Shimadzu Corporation, Kyoto City, Kyoto Prefecture, Japan) at an acceleration voltage of 15 kV to observe the distribution morphology and composition of inclusions.

#### 2.3.6. SEM Analysis of Fracture Surfaces

Using a hand saw, the tensile fracture and impact fracture surfaces were cut from the specimens. The fracture surfaces were placed in a TUC-6H ultrasonic cleaner for 2 min at a cleaning temperature of 45 °C. Subsequently, the fracture surface morphology was observed using a scanning electron microscope (Talos F200x, Thermo Fisher Scientific, Waltham, MA, USA) at an acceleration voltage of 5 kV and a beam current of 0.8 nA, with a working distance (WD) of 22.73 mm.

It should be noted in particular that in this article, the tensile tests and the impact tests at each temperature were all repeated three times.

## 3. Results and Analysis

### 3.1. Metallographic Analysis

**Figure 6:**
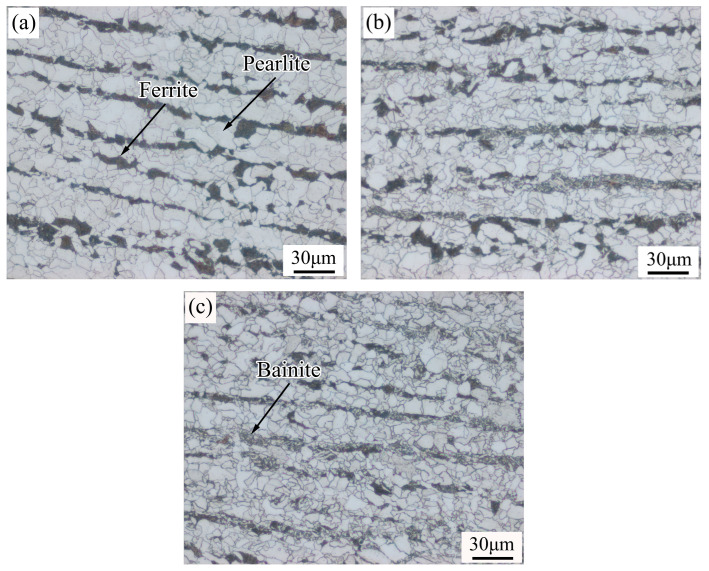
Metallographic images at different Ni contents: (**a**) without Ni addition; (**b**) 0.3 wt%; (**c**) 0.6 wt%.

Figure 6 presents metallographic images of the steels with different Ni contents. As shown, the microstructure undergoes significant changes with increasing Ni content. In the absence of Ni (Figure 6a), the microstructure consists of pearlite and ferrite. With Ni additions of 0.3% and 0.6% (Figure 6b and Figure 6c, respectively), bainite appears alongside pearlite and ferrite, exhibiting a needle-like morphology, and its volume fraction increases with higher Ni content. These changes are attributed to the following factors: Ni expands the austenite phase region during phase transformation, stabilizes undercooled austenite, and partially reduces the driving force for transformation, thereby lowering the austenite-to-ferrite transformation temperature. Additionally, water quenching after rolling accelerates cooling, further depressing the transformation temperature. The combined effects of these two factors promote bainite formation.

**Figure 7:**
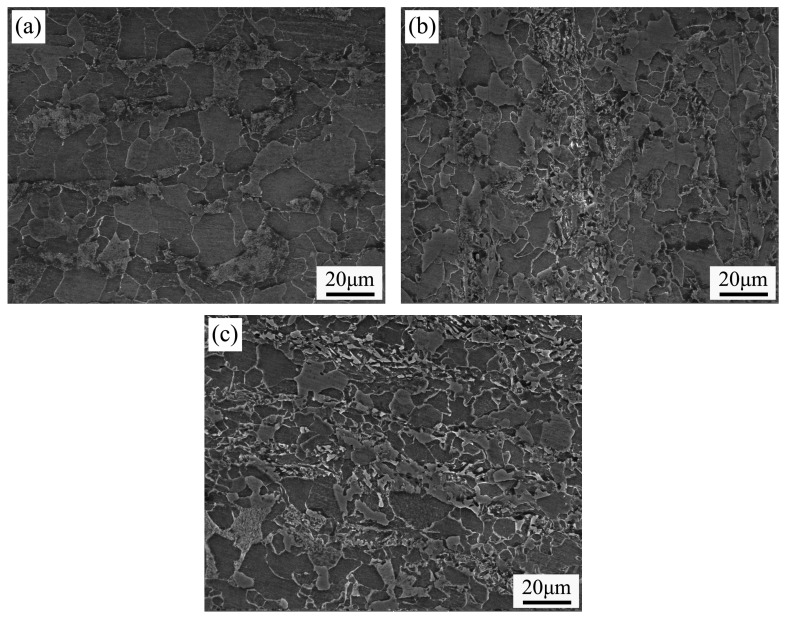
Scanning images at different Ni contents: (**a**) without Ni addition; (**b**) 0.3 wt%; (**c**) 0.6 wt%.

To further analyze the bainite morphology and its specific types, the metallographic specimens were examined using scanning electron microscopy (SEM), as shown in Figure 7. When the Ni content was 0.3%, lath-shaped and fine block-like structures were observed within the microstructure. Based on the relevant literature [21], these structures were identified as upper bainite. When the Ni content was increased to 0.6%, the microstructure consisted of blocky and granular phases appearing to be grayish-white. According to the rolling process described in this study, this microstructure was characterized as granular bainite. The reason for the grayish-white appearance is as follows: during etching with nitric acid alcohol solution, bainitic ferrite is more susceptible to corrosion and becomes relatively recessed, thus appearing gray-black under secondary electron imaging in SEM. In contrast, the austenite phase is less corroded and remains relatively elevated, resulting in a gray-white appearance under secondary electron imaging.

Table 3 presents the grain size statistics for various Ni contents. As shown in the table, the grain size number increases progressively with higher Ni content. Without Ni addition, the grain size number is only 6.8, whereas it increases to 9.2 when the Ni content reaches 0.6%. The grain size number is inversely related to the actual grain size—a higher grain size number corresponds to a smaller average grain size. This indicates that increasing the Ni content promotes grain refinement.

The primary reasons for this phenomenon are twofold. First, the addition of Ni increases the degree of supercooling in Q460 steel. A greater degree of supercooling enhances the nucleation rate during phase transformation and slows down grain growth. Second, Ni can interact with other alloying elements in the steel to form complex compounds or solid solutions, which further impedes grain growth. To verify this conclusion, EPMA was performed on the inclusions in Q460 steel.

### 3.2. EPMA Inclusion Analysis

**Figure 8:**
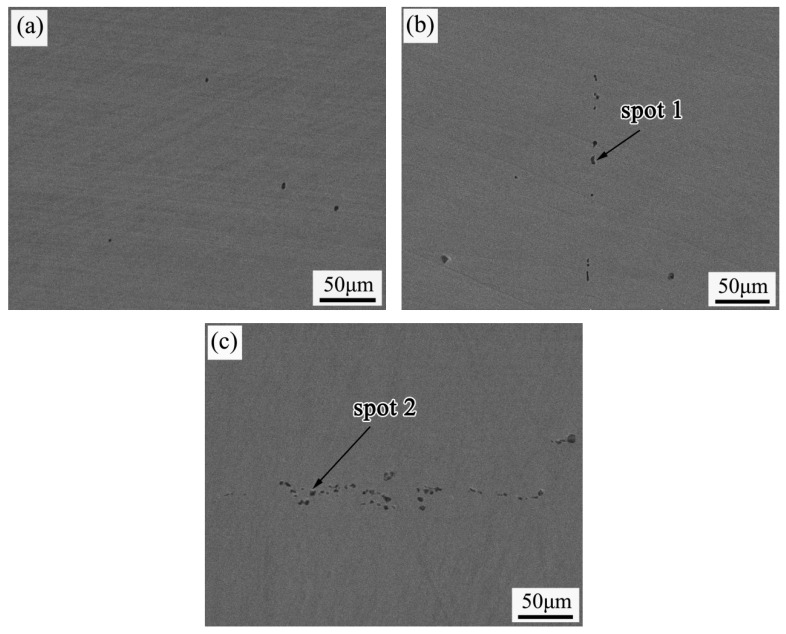
EPMA images of different Ni contents: (**a**) without Ni addition; (**b**) 0.3 wt%; (**c**) 0.6 wt%.

Figure 8 shows the EPMA images for different Ni contents. The distribution of inclusions changes significantly with increasing Ni content. Without the addition of Ni, the inclusions are granular, few in number, and uniformly dispersed. When the Ni content increases to 0.3%, the number of inclusions increases noticeably, with some exhibiting an elongated morphology. As the Ni content is further increased, the number of inclusions rises again, showing a linear distribution and a mixed form of spherical and elongated shapes.

To quantitatively analyze the density and length of the inclusions, the number and size distribution of inclusions within an area of 1 mm^2^ were statistically analyzed, as detailed in Table 4. As shown in the table, both the number and size of inclusions increase continuously with higher Ni content. Without Ni addition, the inclusion density is only 12 per mm^2^, and all lengths fall within the 0–2 μm range. When the Ni content reaches 0.3%, the inclusion density increases to 36 per mm^2^, with lengths mainly concentrated in the 2–4 μm range, and the number of inclusions larger than 4 μm is four. Upon further increasing the Ni content to 0.6%, the inclusion density reaches 85 per mm^2^, and the number of inclusions larger than 4 μm exceeds 30. These results successfully validate the conclusion drawn from the metallographic analysis, that Ni tends to form inclusions, thereby inhibiting grain growth.

To analyze the inclusion types, point scans were performed on selected inclusions. The results are shown in Table 5. Table 5 indicates that Point 1 primarily consists of Ni (22.4%), Al (34.5%), and O (43.1%), while Point 2 mainly comprises Ni (18.6%), Cr (31.5%), and O (49.9%). Based on these data, the predominant inclusion types in Q460 steel plates are Ni-Al oxides and Ni-Cr oxides.

### 3.3. Tensile Properties Analysis

Table 6 presents the tensile property test results for different Ni contents. As shown in the table, with increasing Ni content, the yield strength, tensile strength, and elongation of Q460 steel each exhibit an increasing trend. Without Ni addition, Q460 steel exhibits yield strength, tensile strength, and elongation of 472 MPa, 592 MPa, and 23%, respectively. When Ni content increases to 0.6%, these three properties rise to 552 MPa, 686 MPa, and 30%, respectively. Two primary factors explain these phenomena: First, increased Ni content reduces grain size. Finer grains increase the number of grain boundaries per unit volume. As barriers to dislocation movement, these boundaries enhance the material’s resistance to deformation stress. Second, the finer grains distribute deformation more uniformly across multiple grains, reducing localized stress concentrations. This delays crack initiation and propagation, thereby enhancing plasticity. Second, as Ni content increases, Q460 steel transforms from ferrite to bainite. Compared to ferrite, bainite exhibits higher strength.

Table 6 also reveals that when the Ni content is increased from 0.3 wt% to 0.6 wt%, elongation rises only from 28% to 30%. The primary reason for this phenomenon is that while increased Ni refines grains, it also leads to more inclusions. During tensile testing, these inclusions readily form stress concentrations, causing premature fractures and reduced plasticity.

Surprisingly, when compared to Chinese national standard GB/T 1591-2018, the yield strength, tensile strength, and elongation of Q460 steel at Ni contents of 0.3 wt% and 0.6 wt% significantly exceed the specified values of 460 MPa, 550 MPa, and 18% set by GB/T 1591-2018.

**Figure 9:**
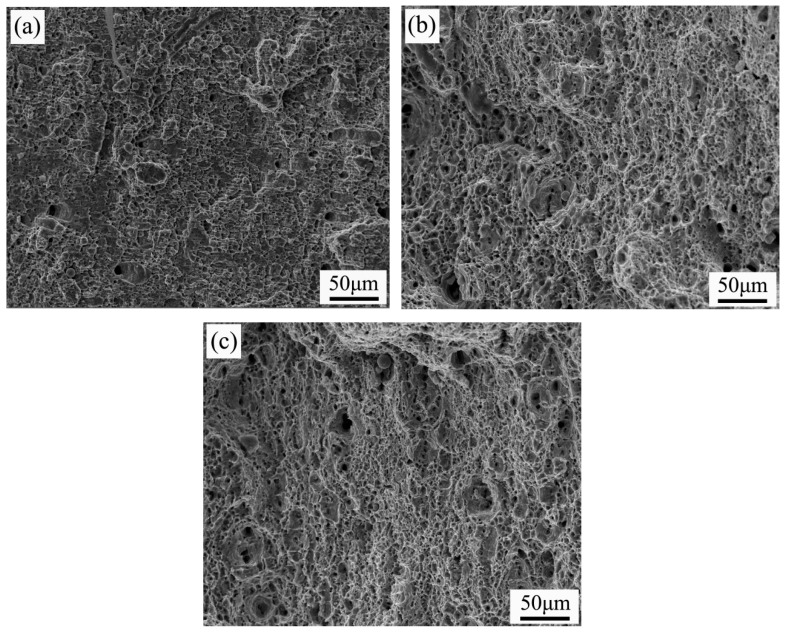
Tensile fracture morphology at different Ni contents: (**a**) without Ni addition; (**b**) 0.3 wt%; (**c**) 0.6 wt%.

Figure 9 presents the fracture morphology of specimens with different Ni contents. In the absence of Ni addition, the fracture surface exhibits small, shallow ductile dimples along with a certain number of cleavage planes and localized shear bands, indicative of a ductile–brittle mixed fracture mode. When the Ni content is increased to 0.3 wt% and 0.6 wt%, the fracture surface is characterized by large, deep, and uniformly distributed ductile dimples with no distinct tearing ridges and demonstrating typical ductile fractures. Notably, as the Ni content rises, a certain quantity of inclusions is observed within the ductile dimples. EDS analysis identifies these inclusions as Ni-Cr-Al mixed oxides.

### 3.4. Impact Performance Analysis

#### 3.4.1. Conventional Impact Performance Analysis

Table 7 presents the conventional impact test results for specimens with different Ni contents. As shown, without Ni addition, the impact energy of the Q460 steel measured 78.2 J at −40 °C and 16.5 J at −60 °C. When the Ni content was 0.3%, the impact energy increased to 145.6 J at −40 °C and 102.1 J at −60 °C, representing an improvement of approximately 80 J. This indicates a sharp rise in impact toughness with increasing Ni content. The primary reason for this improvement is attributed to the grain refinement and increased grain-orientation differences caused by Ni addition, which promote slip coordination across adjacent grains, thereby delaying crack initiation and enhancing impact performance.

It is noteworthy, however, that with a further increase in Ni content to 0.6%, the impact energy reached only 160.5 J at −40 °C and 115.6 J at −60 °C, corresponding to an increment of merely about 15 J—significantly lower than the previous 80 J improvement. This diminished enhancement is explained by the increased inclusion density that accompanies higher Ni content. During impact loading, these inclusions readily act as stress concentrators, reducing the material’s plasticity and consequently degrading its impact resistance.

According to Chinese national standard GB/T 1591-2018, Q460 steel with Ni contents of 0.3% and 0.6% both not only meet the Grade E impact requirements but also substantially exceed the 16 J specified for Grade F at −60 °C.

**Figure 10:**
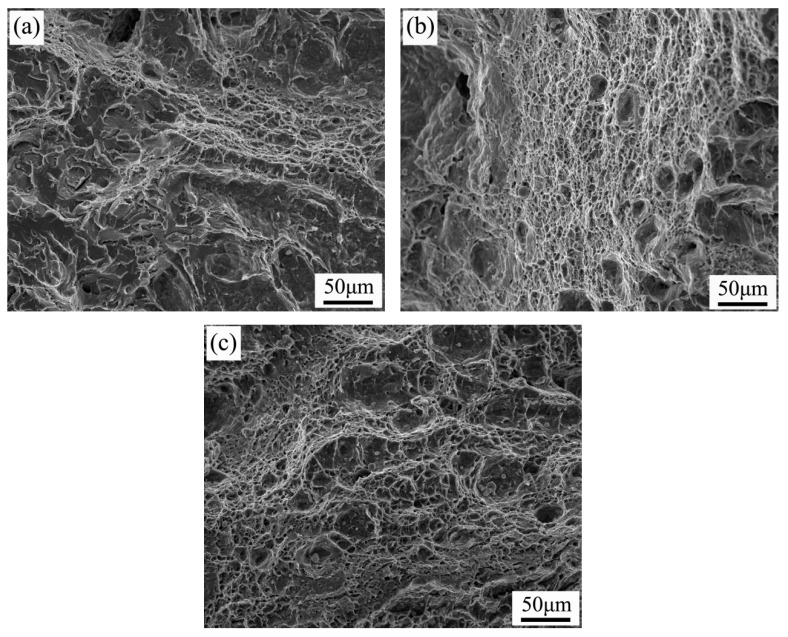
Fracture surface morphology at −40 °C for specimens with different Ni contents: (**a**) without Ni addition; (**b**) 0.3 wt%; (**c**) 0.6 wt%.

Figure 10 shows the fracture morphologies from conventional impact tests at −40 °C for specimens with different Ni contents. Distinct fracture patterns emerge as the Ni content varies. In the absence of Ni addition, fewer dimples are observed, and their distribution is uneven; meanwhile, a certain number of cleavage steps form “river patterns” at different locations. At 0.3% Ni content, the number of ductile dimples increases significantly and becomes more uniformly distributed. Most dimples are circular in shape and small in size, measuring approximately 4–8 μm. It is particularly important to note that a larger number of uniformly distributed dimples indicates better material plasticity. These observations demonstrate that the addition of Ni improves material plasticity. At 0.6% Ni, the fracture surface exhibits large, deep ductile dimples without distinct cleavage planes, characteristic of plastic fracture. However, compared with the Ni-free specimen, this fracture surface shows a significantly increased distribution of inclusions, with maximum inclusion sizes reaching about 10 μm. This observation aligns with the impact performance analysis presented earlier.

#### 3.4.2. Aging Impact Performance Analysis

Table 8 presents the aging impact performance results for the specimens with different Ni contents. As shown, both the −40 °C and −60 °C aging impact energies increase with rising Ni content. Without Ni addition, the aging impact energies were the lowest, measuring 142.6 J at −40 °C and 82.6 J at −60 °C. At 0.6 wt% Ni content, the aging impact energies reached their maximum values of 240.1 J and 182.6 J at −40 °C and −60 °C, respectively, which is consistent with the trend observed in conventional impact tests. When the Ni content increased from 0.3 wt% to 0.6 wt%, the rise in aging impact energy was not pronounced, amounting to only about 10 J. The underlying reasons for this behavior have been detailed in Section 3.4.1 and are not repeated here.

Furthermore, a comparison between Table 7 and Table 8 shows that the Q460 steel exhibits higher impact energy in the aging tests than in the conventional tests, at both −40 °C and −60 °C. This difference is attributed to variations in sampling orientation and testing methodology: conventional impact tests employ transverse specimens, whereas aging impact tests use longitudinal specimens taken after stretching and normalizing.

## 4. Discussion

In this study, it was found that the addition of elemental Ni promotes the formation of bainite. This conclusion aligns with the research of Bagheri et al. [22] in their study on hypoeutectoid steel. Bagheri et al. observed that under the influence of Ni, austenite transformation occurs in two stages. The first stage involves the formation of proeutectoid ferrite, which preferentially nucleates at austenite grain boundaries and inclusions during the phase transformation. The second stage sees the substantial formation of bainite within the ferrite matrix and at fine cementite particles. Krbata et al. [23], in their research on 16Mo3 steel, also found that the mechanical properties of materials are closely related to their microstructure, with significant differences in mechanical properties found among samples with different microstructural constituents.

Furthermore, this study confirms that as the Ni content increases, the number of inclusions rises significantly and their size gradually increases. The primary type of inclusions identified was oxides. This is attributed to the oxygen affinity of Ni; when deoxidation of the molten steel is incomplete, Ni tends to combine with oxygen to form oxides, which precipitate during the cooling stage. Li et al. [24], in their study on stainless steel, found that with increasing Ni and Al content, the number of inclusions in continuous cast slabs increased. EDS analysis revealed that these inclusions were oxides. It is important to note specifically that the grain size of Q460 steel continuously decreases with increasing Ni content. Based on the calculated ASTM grain size numbers, the grain size number was only 6.8 without Ni addition, while it increased to 9.2 when the Ni content was 0.6%. This effective reduction in grain size significantly enhances both the strength and plasticity of the material.

Jana et al. [25], in their research on high-strength steel, discovered that different heat treatment methods substantially influence material properties. Therefore, in the future, besides modifying Ni content to improve the strength and plasticity of Q460 steel, achieving this through various heat treatment processes is also a viable approach that warrants further investigation in subsequent studies.

## 5. Conclusions

(1) With increasing Ni content, undercooled austenite becomes more stable, leading to a microstructural transition in Q460 steel from ferrite + pearlite to bainite + pearlite + ferrite. Furthermore, the grain size progressively decreases with the addition of higher levels of Ni, which is attributed to the lowered phase transformation temperature and the increased amounts of internal precipitates. EPMA point analysis identified the predominant types of precipitates as Ni–Al oxides and Ni–Cr oxides.
(2) As the Ni content rises from 0 wt% to 0.6 wt%, both the yield strength and tensile strength of Q460 steel are significantly enhanced. This improvement results from grain refinement and the extensive formation of bainite.
(3) When the Ni content is increased from 0.3 wt% to 0.6 wt%, the elongation, conventional impact energy, and aged impact energy of Q460 steel exhibit only limited improvement. This is due to the higher inclusion content promoted by the increased Ni addition, which partially deteriorates plasticity.
(4) Taking both cost and performance into consideration, the optimal Ni content for Q460 steel is determined to be 0.3 wt%. At this level, all mechanical properties of Q460 steel substantially exceed the requirements specified in the relevant national standard.

## Figures and Tables

**Table 1:** Specific chemical compositions of ingots (wt%).

Number	C	Si	Mn	Ni	Nb	V	Cr	Als
1	0.180	0.302	1.54	\	0.015	0.021	0.008	0.0
2	0.189	0.301	1.55	0.3	0.016	0.021	0.008	0.02
3	0.180	0.301	1.55	0.6	0.015	0.020	0.008	0.02

**Table 2:** Reduction amount per rolling pass.

Rolling Pass Number	1	2	3	4	5	6	7
Slab thickness (mm)	130	100	80	60	45	30	20

**Table 3:** Grain size data for different Ni contents.

**Ni Content**	0 wt%	0.3 wt%	0.6 wt%
**Grain size**	6.8	8.3	9.2

**Table 4:** Inclusion density and size distribution for different Ni contents.

Ni Content	Density (No./mm^2^)	Size Distribution (No.)		
		0~2 μm	2~4 μm	>4 μm
0 wt%	12	12	0	0
0.3 wt%	36	11	21	4
0.6 wt%	85	8	45	32

**Table 5:** Inclusion composition types (wt%).

	Ni	Al	Cr	O
Spot1	22.4	34.5	—	43.1
Spot2	18.6	—	31.5	49.9

**Table 6:** Tensile properties test results for different Ni content (average ± standard).

Ni Content	Yield Strength (MPa)	Tensile Strength (MPa)	Elongation (%)
0 wt%	472 ± 1.6	592 ± 1.5	23 ± 0.4
0.3 wt%	519 ± 0.8	641 ± 0.7	28 ± 0.3
0.6 wt%	552 ± 1.7	686 ± 3.3	30 ± 0.4

**Table 7:** Conventional impact performance results for different Ni content levels (average ± standard).

Ni Content	−40 °C	−60 °C
0 wt%	78.2 J ± 1.6 J	16.5 J ± 0.4 J
0.3 wt%	145.6 J ± 2.4 J	102.1 J ± 1.8 J
0.6 wt%	160.5 J ± 3.3 J	115.6 J ± 2.6 J

**Table 8:** Ageing impact performance results at different Ni content levels (average ± standard).

Ni Content	−40 °C	−60 °C
0 wt%	142.6 J ± 3.2 J	82.6 J ± 1.5 J
0.3 wt%	223.5 J ± 2.5 J	174.5 J ± 2.1 J
0.6 wt%	235.1 J ± 4.3 J	185.6 J ± 3.5 J

## Data Availability

The original contributions presented in this study are included in the article. Further inquiries can be directed to the corresponding author.

## References

[1] Zhao Q.L., Yan Z.F., Wang S.B., Zhang J., Zhang H., He X., Wang Z., Zhang H., Wang W. (2023). Study on fatigue temperature evolution and failure behavior of Q460 steel. J. Mater. Res. Technol..

[2] Yin X.H., Xu F., Min C.Y., Cheng Y.F., Dong X., Cui B., Xu D. (2021). Promoting the bonding strength and abrasion resistance of brazed diamond using Cu-Sn-Ti composite alloys reinforced with tungsten carbide. Diam. Relat. Mater..

[3] Wang H.Y., Han R.B., Zhang Z.X., Zhu M.L., Liu L.M. (2019). Riveting welding hybrid bonding of high-strength steel and aluminum alloy. Mater. Manuf. Process.

[4] Xue X., Shi Y., Zhou X., Wang J., Xu Y. (2023). Experimental study on the properties of Q960 ultra–high–strength steel after fire exposure. Structures.

[5] Sui Y.H., Zhang X.Z., Guo L., Xu D.F. (2020). Microstructural Study of High Temperature Creep in Q460E Steel Based on the Solidification Method. Trans. Famena.

[6] Gao X.G., Lan H.F., Du L.X., Qiu C.L. (2012). Development of Reduced Rolling Process for Steel Plate. Mater. Sci. Forum.

[7] Miao H., Mei Q., Yuan J.Y., Zheng Z.X., Jin Y., Zuo D. (2016). Low cycle fatigue and strengthening mechanism of cold extruded large diameter internal thread of Q460 steel. Chin. J. Mech. Eng..

[8] Meng Y.J., Wang B., Liu Y.X., Chen M., Dong Z. Researching on Rolling Technology of Q460E Plate. Proceedings of the 4th International Conference on Advanced Composite Materials and Manufacturing Engineering.

[9] Chen Z.Y., Zhao X.J., Qi J.J., Zhu W., Feng Y., Chen L., Wang G. (2022). Effect of tempering on the microstructure and properties of a new multi-functional 460 MPa Grade construction structural steel. J. Mater. Res. Technol..

[10] Wang L., Gao C.R., Wang Y.F., Jin W.Z., Zhao D.-W., Liu X.-H. (2010). Effect of Thermomechanical Controlled Processing Parameters on Microstructure and Properties of Q460q Steel. J. Iron Steel Res. Int..

[11] Yuan S.F., Shang C.J., Xie Z.J., Wang X.M., Yang J.R., Misra R.D.K. (2018). Impact of Intercritical Annealing on Retained Austenite and Toughness of a 460 MPa Grade Multiphase Heavy Gauge Plate Steel. Steel Res. Int..

[12] Guo L., Sui Y.H., Zhang X.Z. (2018). High-temperature creep constitutional model of Q460E steel and effect of creep on bulging deformation of continuous casting slab. J. Iron Steel Res. Int..

[13] Liu L.L., Zhang H., Bi H., Chang E., Li M. (2025). CInfluence of alloying elements on the microstructure and pitting behavior of high-strength Cr-Mn-Ni-N metastable austenitic stainless steels in the atmospheric environments with chloride ions. Mater. Charact..

[14] Mohrbacher H., Kern A. (2023). Nickel Alloying in Carbon Steel: Fundamentals and Applications. Alloys.

[15] Qian Y., Yu L.Z., Fei Y.Z. (2024). Influence of Nickel on Microstructure and Mechanical Properties in Medium-Carbon Spring Steel. Materials.

[16] Kim K., Lee S.-J. (2017). Effect of Ni addition on the mechanical behavior of quenching and partitioning (Q&P) steel. Mater. Sci. Eng. A.

[17] Qin Z.H., He D.Y., Ma L.X., He C.X., Wu X., Wang G. (2024). Influence of Ni additions on microstructure, non-magnetic properties, and wear resistance of Fe–Mn-Cr alloy deposited by metal-cored arc welding. Weld. World.

[18] Liu Z.P., Yu Y.S., Wang Z.Q., Shang C.J. (2022). Morphology and Crystallography Analyses of HSLA Steels with Hardenability Enhanced by Tailored C–Ni Collocation. Metals.

[19] (2018). Low Alloy High Strength Structural Steel.

[20] (2017). Method for Determining the Average Grain Size of Metals.

[21] Teagho F.T., Sennour M., Maziere M., Galtier A., Gourgues-Lorenzon A.-F. (2026). Cleavage fracture of high strength tempered martensite and mixed tempered martensite  + upper bainite medium carbon steel. Int. J. Fract..

[22] Bagheri Y., Kamali H., Kamali E., Nedjad S.H. (2022). Formation of nodular bainite in an Fe-9.10Ni-0.06C (wt. %) alloy: A new microstructure for cryogenic steels. Scr. Mater..

[23] Krbata M., Ciger R., Kohutiar M., Sozańska M., Eckert M., Barenyi I., Kianicova M., Jus M., Beronská N., Mendala B. (2023). Effect of Supercritical Bending on the Mechanical & Tribological Properties of Inconel 625 Welded Using the Cold Metal Transfer Method on a 16Mo3 Steel Pipe. Materials.

[24] Li J.Y., Cheng G.G., Li L.Y., Hu B., Xu C., Wang G. (2019). Formation Mechanism of Oxide Inclusions in Cr-Mn-Ni Stainless Steel. ISIJ Int..

[25] Escherová J., Krbat’A M., Klučiar P., Jus M., Chochlíková H., Daniel K., Polášek M. (2025). The Influence of Heat Treatment of High-Strength Steels on the Change of Tribological Properties by the Ball on Flat Method. Key Eng. Mater..

